# Crystal structure of *N*-[4-amino-5-cyano-6-(methyl­sulfan­yl)pyridin-2-yl]-2-chloro­acetamide

**DOI:** 10.1107/S2056989015002431

**Published:** 2015-02-13

**Authors:** Shaaban K. Mohamed, Kyle S. Knight, Mehmet Akkurt, Bahgat R. M. Hussein, Mustafa R. Albayati

**Affiliations:** a Chemistry and Environmental Division, Manchester Metropolitan University, Manchester M1 5GD, England; b Chemistry Department, Faculty of Science, Minia University, 61519 El-Minia, Egypt; c Department of Chemistry, The University of Tennessee at Chattanooga, Chattanooga, TN 37403, USA; d Department of Physics, Faculty of Sciences, Erciyes University, 38039 Kayseri, Turkey; e Chemistry Department, Faculty of Science, Sohag University, 82524 Sohag, Egypt; f Kirkuk University, College of Science, Department of Chemistry, Kirkuk, Iraq

**Keywords:** crystal structure, *N*-[4-amino-5-cyano-6-(methyl­sulfan­yl)pyridin-2-yl]-2-chloro­acetamide, polyfunctional pyridines, hydrogen bonding

## Abstract

In the title compound, C_9_H_9_ClN_4_OS, the dihedral angle between the acetamide moiety and the pyridine ring is 4.83 (12)°. The O=C—C—Cl torsion angle is 46.4 (3)° and an intra­molecular C—H⋯O inter­action generates an *S*(6) ring. In the crystal, mol­ecules are linked by N—H⋯O, N—H⋯N and C—H⋯N hydrogen bonds, generating sheets lying parallel to (120).

## Related literature   

For medicinal and industrial applications of pyridine-containing compounds, see: Boger & Nakahara (1991 ▸); Zhang *et al.* (1995 ▸); Castedo *et al.* (1984 ▸); Latif *et al.* (1981 ▸), Mamolo *et al.* (2001 ▸); Gachet *et al.* (1995 ▸).
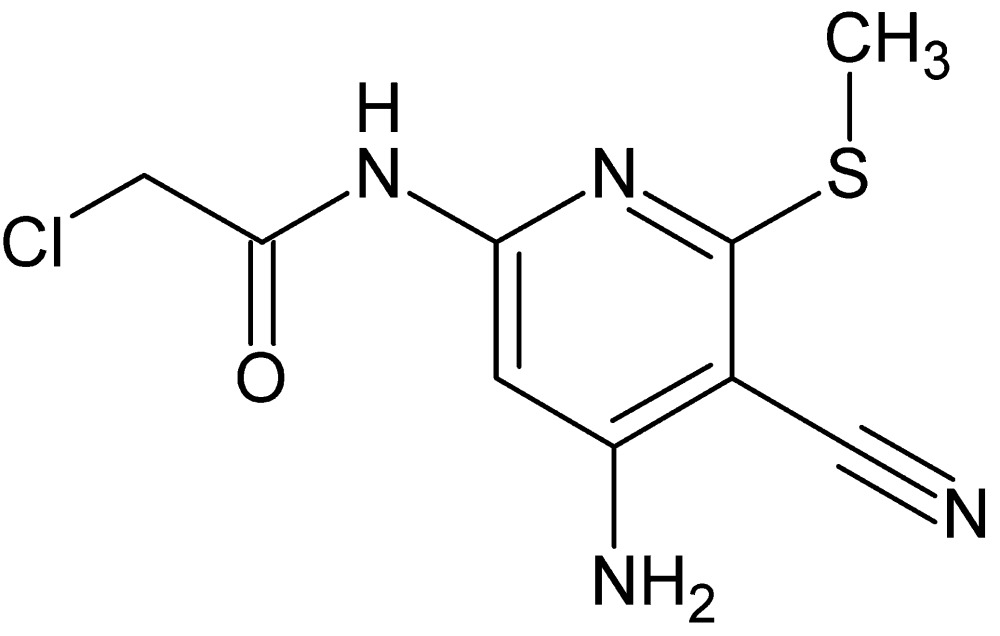


## Experimental   

### Crystal data  

C_9_H_9_ClN_4_OS
*M* *_r_* = 256.71
Monoclinic, 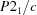

*a* = 5.1654 (7) Å
*b* = 20.483 (3) Å
*c* = 10.9489 (14) Å
β = 103.562 (5)°
*V* = 1126.1 (3) Å^3^
*Z* = 4
Mo *K*α radiation
μ = 0.51 mm^−1^
*T* = 200 K
0.50 × 0.16 × 0.10 mm

### Data collection  

Bruker SMART X2S benchtop diffractometer
Absorption correction: multi-scan (*SADABS*; Bruker, 2008 ▸) *T*_min_ = 0.756, *T*_max_ = 0.951
11998 measured reflections
1960 independent reflections
1634 reflections with *I* > 2σ(*I*)
*R*_int_ = 0.043

### Refinement  

*R*[*F* ^2^ > 2σ(*F* ^2^)] = 0.035
*wR*(*F* ^2^) = 0.096
*S* = 1.05
1960 reflections
152 parameters
3 restraints
H atoms treated by a mixture of independent and constrained refinement
Δρ_max_ = 0.34 e Å^−3^
Δρ_min_ = −0.41 e Å^−3^

### 

Data collection: *APEX2* (Bruker, 2009 ▸); cell refinement: *SAINT* (Bruker, 2009 ▸); data reduction: *SAINT*; program(s) used to solve structure: *SHELXS2014* (Sheldrick, 2008 ▸); program(s) used to refine structure: *SHELXL2014* (Sheldrick, 2015 ▸); molecular graphics: *ORTEP-3 for Windows* (Farrugia, 2012 ▸); software used to prepare material for publication: *PLATON* (Spek, 2009 ▸).

## Supplementary Material

Crystal structure: contains datablock(s) global, I. DOI: 10.1107/S2056989015002431/hb7362sup1.cif

Structure factors: contains datablock(s) I. DOI: 10.1107/S2056989015002431/hb7362Isup2.hkl

Supporting information file. DOI: 10.1107/S2056989015002431/hb7362Isup3.cml

**Fig. 1.**
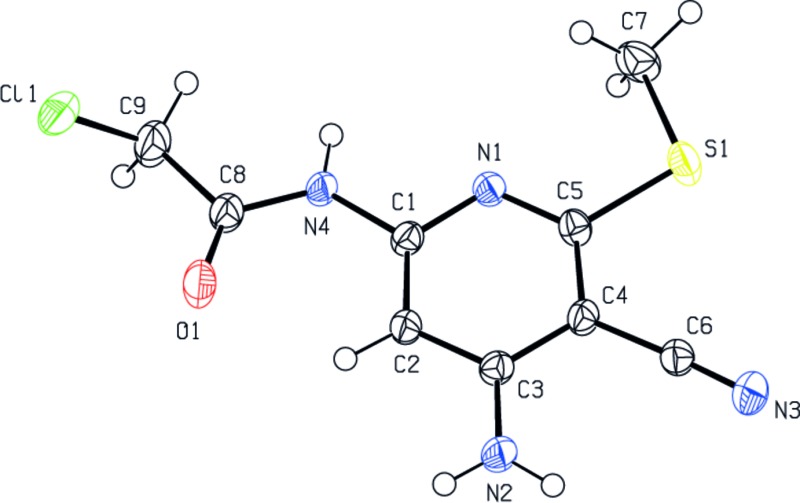
Perspective view of the title compound with displacement ellipsoids drawn at the 50% probability level.

**Fig. 2.**
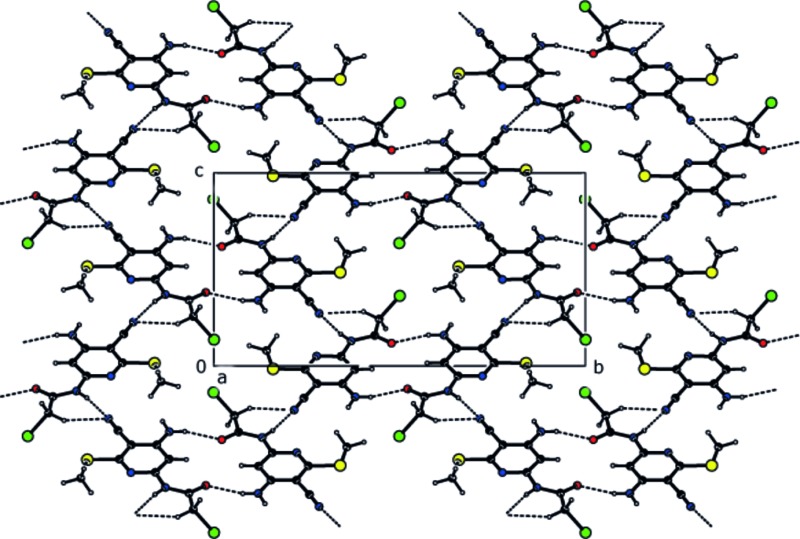
View of the hydrogen bonding and packing of the title compound viewed along the a axis.

**Fig. 3.**
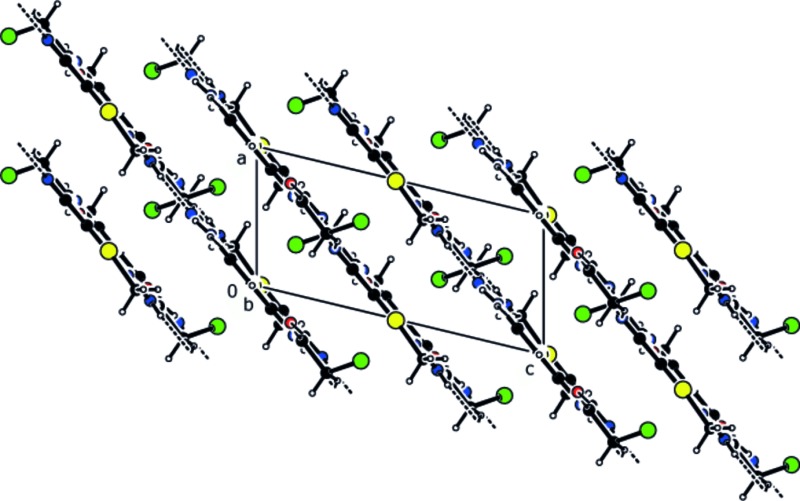
Packing of the title compound viewed along the b axis.

. DOI: 10.1107/S2056989015002431/hb7362fig1.tif
Perspective view of the title compound with displacement ellipsoids drawn at the 50% probability level.

a . DOI: 10.1107/S2056989015002431/hb7362fig2.tif
View of the hydrogen bonding and packing of the title compound viewed along the *a* axis.

b . DOI: 10.1107/S2056989015002431/hb7362fig3.tif
Packing of the title compound viewed along the *b* axis.

CCDC reference: 1047552

Additional supporting information:  crystallographic information; 3D view; checkCIF report

## Figures and Tables

**Table 1:** Hydrogen-bond geometry (, )

*D*H*A*	*D*H	H*A*	*D* *A*	*D*H*A*
C2H2O1	0.95	2.26	2.863(3)	121
N2H2*B*O1^i^	0.86(1)	2.13(1)	2.968(2)	165(3)
N4H4N3^ii^	0.88	2.16	3.034(2)	172
C9H9*B*N3^ii^	0.99	2.47	3.366(3)	151

Symmetry codes: (i) 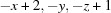; (ii) 
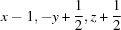
.

## supplementary crystallographic information

### S1. Comment

Pyridine compounds have occupied a unique position in medicinal and industrial chemistry. Some polyfuctional pyridines constitute an important class of antitumor compounds (Boger & Nakahara, 1991; Zhang *et al.*, 1995). They also show antibacterial (Castedo *et al.*, 1984), antifungal (Latif *et al.*, 1981), antimyotic (Mamolo *et al.*, 2001) and antidepressant (Gachet *et al.*, 1995) activities. In this respect, and also in continuation of our study on synthesis of different heterocyclic system that containing highly biological activity, we report here the synthesis and crystal structur of the title compound.

In the title compound (Fig. 1), the chloroacetamide moiety has an extended conformation, as indicated by the torsion angles around the C8—N4 [C9—C8—N4—C1 = 177.90 (19)°] and N4—C1 [C8—N4—C1—C2 = 4.0 (3)°] bonds. The sum of the angles around atom N4 (360.28°) suggests *sp*^2^-hybridization. The dihedral angle between the pyridine ring (N1/C1–C5) and the chloroacetamide moiety is 22.36 (6)°.

Molecular structure is stabilized by a weak intramolecular C—H···O interaction (Table 1). In the crystal, molecules are linked *via* intermolecular N—H···O, N—H···N and C—H···N hydrogen bonds (Table 1, Figs. 2 & 3), forming two dimensional networks paralel to the (120) planes.

### S2. Experimental

In an ice bath, to a solution of 4,6-Diamino-3-cyano-2-methylthiopyridine-2(1*H*)-thione (0.5 g, 2.7 mmol) in 30 ml dioxane, chloroacetyl chloride (0.31 g, 2.7 mmol) was added drop by drop with stirring at 273 K over 30 min. The resulting solid product was filtered off under vacuum, washed with cold ethanol, dried and recrystallized from ethanol to furnish colourless crystals (yield 0.65 g, 91%) Mp. 529 – 531 K.

### S3. Refinement

H-atoms attached to carbon were placed in calculated positions (C—H = 0.95 - 0.99 Å). They were included as riding contributions with isotropic displacement parameters 1.2 or 1.5 times those of the attached atoms. The hydrogen atoms attached to N2 were found from difference Fourier maps and their *U*_iso_ were refined riding contributions with isotropic displacement parameters 1.5 times those of the attached atom, with N2—H2A = 0.85 (3) and N2—H2B = 0.856 (11) Å.

### Crystal data

**Table:**

C_9_H_9_ClN_4_OS	*F*(000) = 528
*M**_r_* = 256.71	*D*_x_ = 1.514 Mg m^−^^3^
Monoclinic, *P*2_1_/*c*	Mo *K*α radiation, λ = 0.71073 Å
Hall symbol: -P 2ybc	Cell parameters from 3684 reflections
*a* = 5.1654 (7) Å	θ = 2.8–24.9°
*b* = 20.483 (3) Å	µ = 0.51 mm^−^^1^
*c* = 10.9489 (14) Å	*T* = 200 K
β = 103.562 (5)°	Needle, yellow
*V* = 1126.1 (3) Å^3^	0.50 × 0.16 × 0.10 mm
*Z* = 4	

### Data collection

**Table:**

Bruker SMART X2S benchtop diffractometer	1960 independent reflections
Radiation source: XOS X-beam microfocus source	1634 reflections with *I* > 2σ(*I*)
Doubly curved silicon crystal monochromator	*R*_int_ = 0.043
ω scans	θ_max_ = 25.0°, θ_min_ = 2.8°
Absorption correction: multi-scan (*SADABS*; Bruker, 2008)	*h* = −6→6
*T*_min_ = 0.756, *T*_max_ = 0.951	*k* = −24→24
11998 measured reflections	*l* = −12→10

### Refinement

**Table:**

Refinement on *F*^2^	3 restraints
Least-squares matrix: full	Hydrogen site location: mixed
*R*[*F*^2^ > 2σ(*F*^2^)] = 0.035	H atoms treated by a mixture of independent and constrained refinement
*wR*(*F*^2^) = 0.096	*w* = 1/[σ^2^(*F*_o_^2^) + (0.0489*P*)^2^ + 0.4578*P*] where *P* = (*F*_o_^2^ + 2*F*_c_^2^)/3
*S* = 1.05	(Δ/σ)_max_ < 0.001
1960 reflections	Δρ_max_ = 0.34 e Å^−^^3^
152 parameters	Δρ_min_ = −0.41 e Å^−^^3^

### Special details

**Table:**

Geometry. Bond distances, angles *etc*. have been calculated using the rounded fractional coordinates. All su's are estimated from the variances of the (full) variance-covariance matrix. The cell e.s.d.'s are taken into account in the estimation of distances, angles and torsion angles
Refinement. Refinement on *F*^2^ for ALL reflections except those flagged by the user for potential systematic errors. Weighted *R*-factors *wR* and all goodnesses of fit *S* are based on *F*^2^, conventional *R*-factors *R* are based on *F*, with *F* set to zero for negative *F*^2^. The observed criterion of *F*^2^ > σ(*F*^2^) is used only for calculating -*R*-factor-obs *etc*. and is not relevant to the choice of reflections for refinement. *R*-factors based on *F*^2^ are statistically about twice as large as those based on *F*, and *R*-factors based on ALL data will be even larger.

### Fractional atomic coordinates and isotropic or equivalent isotropic displacement parameters (Å^2^)

**Table:**

	*x*	*y*	*z*	*U*_iso_*/*U*_eq_	
Cl1	0.63935 (18)	−0.00064 (3)	0.86334 (7)	0.0591 (3)	
S1	0.99138 (10)	0.34034 (2)	0.48516 (6)	0.0319 (2)	
O1	0.7921 (4)	0.02162 (8)	0.61866 (18)	0.0494 (6)	
N1	0.8476 (3)	0.22309 (8)	0.55160 (16)	0.0229 (5)	
N2	1.2836 (4)	0.11962 (8)	0.34016 (18)	0.0302 (6)	
N3	1.4013 (3)	0.28346 (9)	0.27223 (18)	0.0341 (6)	
N4	0.7070 (3)	0.13030 (8)	0.62971 (16)	0.0251 (5)	
C1	0.8574 (4)	0.15744 (9)	0.55067 (18)	0.0220 (6)	
C2	0.9959 (4)	0.12055 (10)	0.48191 (18)	0.0246 (6)	
C3	1.1424 (4)	0.15360 (9)	0.40844 (18)	0.0226 (6)	
C4	1.1368 (4)	0.22219 (10)	0.40856 (19)	0.0228 (6)	
C5	0.9869 (4)	0.25440 (10)	0.48268 (19)	0.0227 (6)	
C6	1.2825 (4)	0.25766 (9)	0.3341 (2)	0.0249 (6)	
C7	0.7603 (4)	0.35817 (11)	0.5806 (2)	0.0355 (7)	
C8	0.6819 (4)	0.06691 (10)	0.6581 (2)	0.0304 (7)	
C9	0.4941 (5)	0.05515 (11)	0.7435 (2)	0.0416 (8)	
H2	0.99210	0.07420	0.48430	0.0300*	
H2A	1.358 (6)	0.1378 (14)	0.287 (2)	0.0890*	
H2B	1.269 (7)	0.0780 (5)	0.339 (3)	0.0890*	
H4	0.61790	0.15830	0.66520	0.0300*	
H7A	0.57850	0.34940	0.53250	0.0530*	
H7B	0.77610	0.40420	0.60550	0.0530*	
H7C	0.80060	0.33060	0.65590	0.0530*	
H9A	0.32330	0.03740	0.69400	0.0500*	
H9B	0.45690	0.09690	0.78160	0.0500*	

### Atomic displacement parameters (Å^2^)

**Table:**

	*U*^11^	*U*^22^	*U*^33^	*U*^12^	*U*^13^	*U*^23^
Cl1	0.1097 (6)	0.0365 (4)	0.0449 (4)	0.0030 (3)	0.0458 (4)	0.0068 (3)
S1	0.0342 (3)	0.0178 (3)	0.0481 (4)	−0.0007 (2)	0.0187 (3)	0.0002 (2)
O1	0.0775 (12)	0.0217 (8)	0.0684 (13)	0.0051 (8)	0.0560 (10)	0.0063 (8)
N1	0.0238 (8)	0.0210 (8)	0.0258 (9)	−0.0004 (6)	0.0098 (7)	−0.0004 (7)
N2	0.0379 (10)	0.0258 (9)	0.0344 (11)	0.0008 (8)	0.0236 (8)	−0.0009 (8)
N3	0.0357 (10)	0.0333 (10)	0.0374 (11)	−0.0070 (8)	0.0168 (9)	0.0024 (9)
N4	0.0313 (9)	0.0204 (8)	0.0299 (10)	0.0014 (7)	0.0198 (7)	0.0009 (7)
C1	0.0225 (9)	0.0230 (10)	0.0221 (11)	0.0007 (8)	0.0086 (8)	0.0021 (8)
C2	0.0299 (10)	0.0201 (10)	0.0274 (11)	−0.0009 (8)	0.0139 (9)	0.0005 (8)
C3	0.0221 (9)	0.0233 (10)	0.0238 (11)	0.0013 (8)	0.0085 (8)	0.0005 (8)
C4	0.0224 (10)	0.0226 (10)	0.0252 (11)	−0.0024 (7)	0.0095 (8)	0.0017 (8)
C5	0.0204 (9)	0.0200 (10)	0.0280 (11)	−0.0008 (7)	0.0063 (8)	0.0004 (8)
C6	0.0263 (10)	0.0223 (10)	0.0275 (11)	−0.0013 (8)	0.0093 (9)	0.0001 (9)
C7	0.0363 (12)	0.0281 (12)	0.0445 (14)	0.0043 (9)	0.0141 (10)	−0.0061 (10)
C8	0.0380 (11)	0.0239 (11)	0.0354 (13)	0.0009 (9)	0.0209 (10)	0.0022 (9)
C9	0.0536 (14)	0.0309 (12)	0.0516 (16)	0.0008 (11)	0.0353 (12)	0.0055 (11)

### Geometric parameters (Å, º)

**Table:**

Cl1—C9	1.769 (2)	N2—H2B	0.856 (11)
S1—C5	1.761 (2)	C3—C4	1.405 (3)
S1—C7	1.799 (2)	N4—H4	0.8800
O1—C8	1.219 (3)	C4—C5	1.410 (3)
N1—C1	1.346 (2)	C4—C6	1.431 (3)
N1—C5	1.324 (3)	C8—C9	1.516 (3)
N2—C3	1.353 (3)	C2—H2	0.9500
N3—C6	1.144 (3)	C7—H7A	0.9800
N4—C1	1.406 (3)	C7—H7B	0.9800
N4—C8	1.348 (3)	C7—H7C	0.9800
C1—C2	1.379 (3)	C9—H9A	0.9900
C2—C3	1.401 (3)	C9—H9B	0.9900
N2—H2A	0.85 (3)		
			
C5—S1—C7	101.76 (10)	S1—C5—C4	118.02 (16)
C1—N1—C5	116.95 (17)	N3—C6—C4	177.0 (2)
C1—N4—C8	128.28 (17)	N4—C8—C9	113.90 (18)
N1—C1—N4	111.30 (17)	O1—C8—N4	125.0 (2)
N1—C1—C2	125.23 (19)	O1—C8—C9	121.1 (2)
N4—C1—C2	123.48 (17)	Cl1—C9—C8	109.80 (17)
C1—C2—C3	117.89 (18)	C1—C2—H2	121.00
C3—N2—H2B	118 (2)	C3—C2—H2	121.00
H2A—N2—H2B	118 (3)	S1—C7—H7A	109.00
C3—N2—H2A	122.6 (19)	S1—C7—H7B	110.00
C2—C3—C4	117.94 (18)	S1—C7—H7C	109.00
N2—C3—C4	121.91 (18)	H7A—C7—H7B	109.00
N2—C3—C2	120.15 (17)	H7A—C7—H7C	110.00
C3—C4—C5	118.85 (19)	H7B—C7—H7C	109.00
C3—C4—C6	119.57 (18)	Cl1—C9—H9A	110.00
C5—C4—C6	121.58 (18)	Cl1—C9—H9B	110.00
C1—N4—H4	116.00	C8—C9—H9A	110.00
C8—N4—H4	116.00	C8—C9—H9B	110.00
S1—C5—N1	118.85 (15)	H9A—C9—H9B	108.00
N1—C5—C4	123.13 (19)		
			
C7—S1—C5—N1	−4.84 (19)	C1—C2—C3—N2	179.40 (19)
C7—S1—C5—C4	176.27 (17)	C1—C2—C3—C4	−0.6 (3)
C5—N1—C1—N4	178.53 (17)	N2—C3—C4—C5	−179.5 (2)
C1—N1—C5—S1	−177.61 (15)	C2—C3—C4—C6	−179.61 (19)
C1—N1—C5—C4	1.2 (3)	N2—C3—C4—C6	0.4 (3)
C5—N1—C1—C2	−1.4 (3)	C2—C3—C4—C5	0.5 (3)
C1—N4—C8—O1	0.6 (4)	C6—C4—C5—N1	179.3 (2)
C8—N4—C1—N1	−175.96 (19)	C3—C4—C5—S1	178.04 (16)
C8—N4—C1—C2	4.0 (3)	C3—C4—C5—N1	−0.8 (3)
C1—N4—C8—C9	−177.90 (19)	C6—C4—C5—S1	−1.9 (3)
N4—C1—C2—C3	−178.82 (18)	O1—C8—C9—Cl1	46.4 (3)
N1—C1—C2—C3	1.1 (3)	N4—C8—C9—Cl1	−135.06 (17)

### Hydrogen-bond geometry (Å, º)

**Table:**

*D*—H···*A*	*D*—H	H···*A*	*D*···*A*	*D*—H···*A*
C2—H2···O1	0.95	2.26	2.863 (3)	121
N2—H2*B*···O1^i^	0.86 (1)	2.13 (1)	2.968 (2)	165 (3)
N4—H4···N3^ii^	0.88	2.16	3.034 (2)	172
C9—H9*B*···N3^ii^	0.99	2.47	3.366 (3)	151

Symmetry codes: (i) −*x*+2, −*y*, −*z*+1; (ii) *x*−1, −*y*+1/2, *z*+1/2.
