# Supplementary figures and images for: Crystal structure of N-[4-amino-5-cyano-6-(methyl­sulfan­yl)pyridin-2-yl]-2-chloro­acetamide
Source: Acta Crystallogr E Crystallogr Commun. 2015 Feb 13;71(Pt 3):o169–70. doi: 10.1107/S2056989015002431 (PMC4350726; doi:10.1107/S2056989015002431)

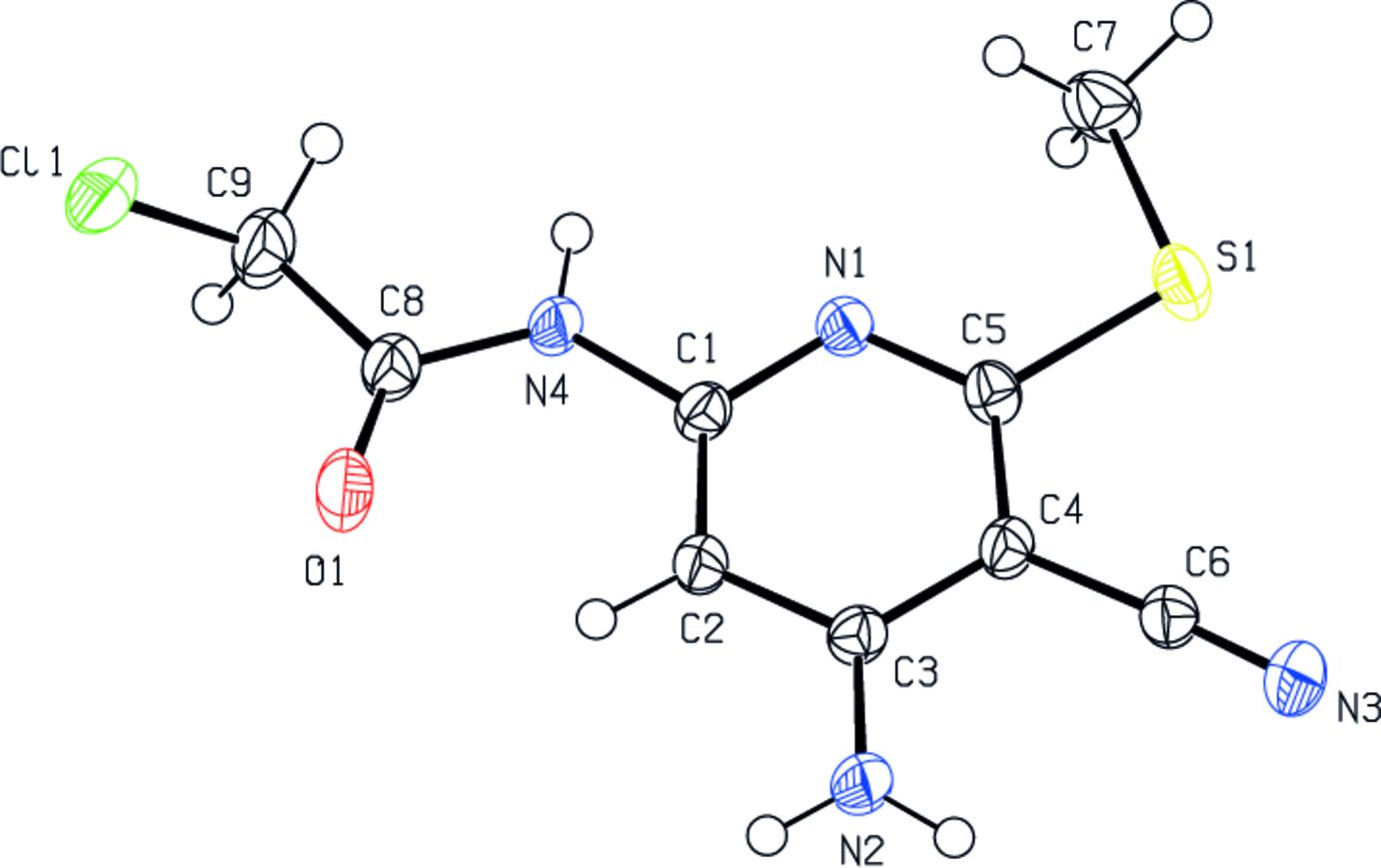

Supplement: Supplementary file 4 [file e-71-0o169-fig1.tif]

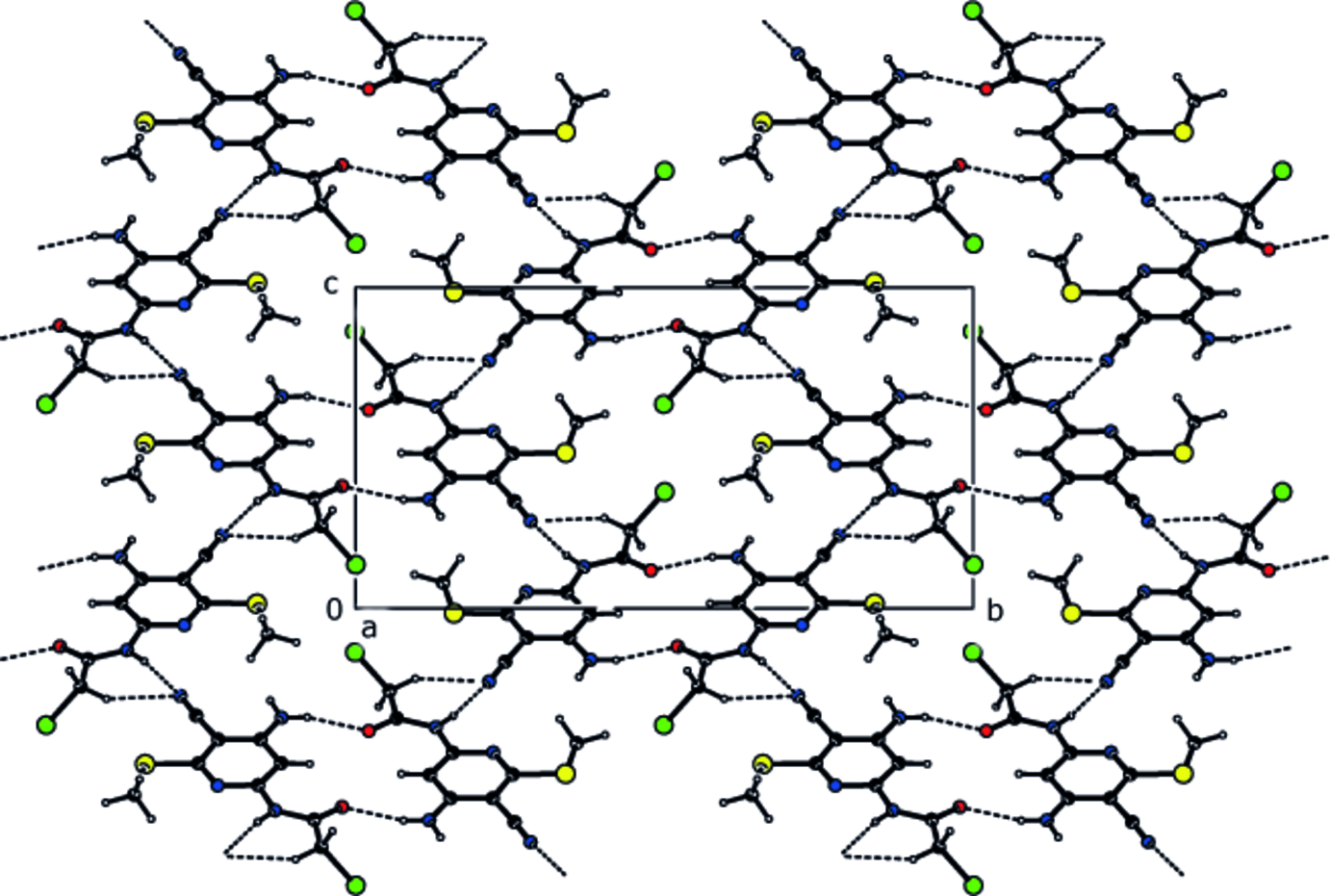

Supplement: Supplementary file 5 [file e-71-0o169-fig2.tif]

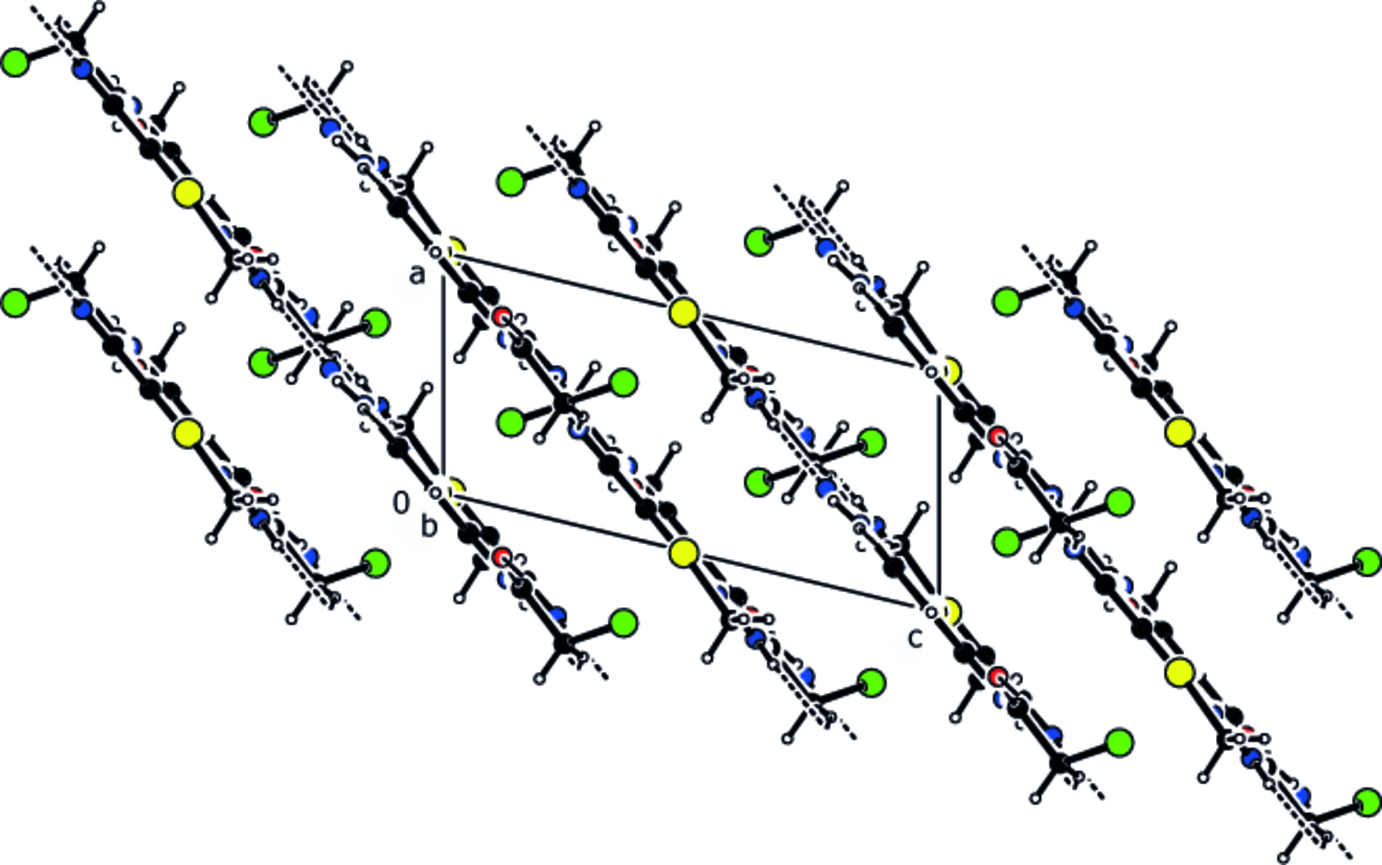

Supplement: Supplementary file 6 [file e-71-0o169-fig3.tif]
